# Exploring Adaptive Health Technology Assessment for Evaluating Ten Cancer Interventions: Insights and Lessons from a Pilot Study in India

**DOI:** 10.1136/bmjebm-2023-112490

**Published:** 2025-11-26

**Authors:** Srobana Ghosh, C S Pramesh, Manju Sengar, Priya Ranganathan, Francis Ruiz, Tabassum Wadasadawala, Prakash Nayak, Jayashree Thorat, Apurva Ashok, Malkeet Singh, Abha Mehndiratta, Cassandra Nemzoff, Hiral Anil Shah

**Affiliations:** 1 Center for Global Development, London, UK; 2 Tata Memorial Centre, Homi Bhabha National Institute, Mumbai, Maharashtra, India; 3 London School of Hygiene and Tropical Medicine, UK

**Keywords:** Cancer, cost effectiveness analysis, health technology assessment, evaluation criteria, health priorities, health resources, National Health Programs, Guidelines as topic

## Abstract

**Background:**

Health technology assessment (HTA) is a valuable tool for informing the efficient allocation of resources in healthcare. However, the resource-intensive nature of HTA can limit its application, especially in low-resource settings. Adapting HTA processes by incorporating available international evidence offers a pragmatic approach to provide evidence for decision-making where resources are constrained.

**Objective:**

This study piloted an adaptive health technology assessment (aHTA) method to evaluate ten cancer interventions.

**Methods:**

We arranged a joint collaboration with the International Decision Support Initiative and the National Cancer Grid in India to form a working group of clinicians and health economists. We conducted a rapid review of HTA reports and economic evaluations for ten prioritised common cancer interventions for breast, lung, and head & neck cancers. We extracted data on cost-effectiveness, conducted a price benchmarking analysis, estimated treatment costs and calculated the treatment’s share of the national insurance family allowance. Finally, we determined through qualitative appraisal whether the intervention was likely to be considered cost-effective in the Indian context.

**Results:**

Of the 10 interventions assessed, 9 had sufficient evidence to make determinations on the likely cost-effectiveness. Three were potentially cost-effective (one after a price discount and another by using the generic price); while five were not, and one was only cost-effective in a subgroup. One intervention required a full HTA due to remaining uncertainty. Information on the likely cost-effectiveness, clinical and safety benefits, and treatment costs was consistently found through publicly available evidence. Assessment methods were modified slightly across the ten interventions, including expanding the data extraction criteria, updating the calculations, and broadening the evidence retrieval.

**Conclusion:**

The aHTA method is a feasible resource-sensitive alternative to traditional HTA for informing decision-making in resource-constrained settings when ample international data on cost-effectiveness for a given topic is available.

## Introduction

India faces a double burden of disease (1, 2): with high rates of both communicable and non-communicable diseases(3). In particular, cancer incidence in India is rising, reaching 1.3 million (4, 5), a concerning trend given the high costs of treatment, which most patients access through the private sector(6, 7, 8).

A commitment to expanding public access to cancer care is underway through a collaboration between the National Cancer Grid (NCG) and the National Health Authority (NHA). The NCG, a network of 287 cancer hospitals (9–11), is the primary developer of clinical practice guidelines for oncology in India (18) and are working with the NHA to link their guidelines to the oncology health benefit packages (HBPs) for the national health insurance scheme Ayushman Bharat Pradhan Mantri Jan Arogya Yojana (AB-PMJAY) (12, 13) which define the available cancer treatments under the national health care plan, increasing access to cancer care for the poorest patients.

Yet India has limited public funding for cancer treatment (<2% of GDP)(6, 14, 15) and there is a need for the AB-PMJAY to sustainably cover all disease areas, both communicable and non-communicable, which necessitates efficient, value-based care, especially given the increasingly exorbitant costs of novel cancer therapies (16, 17). Therefore, the NCG wanted to prioritise only the most cost-effective treatments to be made available under AB-PMJAY(18), which required an objective assessment of cost-effectiveness.

Health technology assessment (HTA) is traditionally used to determine the value of a health intervention(19) through a multidisciplinary process that uses both systematic and explicit methods, including cost-effectiveness analyses(20). While India’s national HTA body (HTAIn)(21, 22) has conducted many studies, national-level topic prioritization did not cover all of the interventions which needed to be evaluated by the NCG. Additionally, resource, capacity and time constraints (23, 24) limited the NCG’s ability to conduct full HTAs, prompting them to explore alternative approaches to incorporate economic evidence to inform decision-making.

A potential solution was to adapt the HTA process, known as adaptive or rapid HTA (aHTA), defined by Nemzoff et al. (25, 26) as a structured approach to selecting and conducting the optimal HTA analysis to produce efficient HTA results by adjusting for analytical time, data, capacity, and source of conduct, by leveraging information from other settings where possible (26).

Several countries use aHTA methods, including rapid reviews, transferring evidence and streamlined economic evaluations (26, 27). However, while aHTA has the potential to improve the efficiency of any priority-setting system, there is no single established method.

In the absence of set methods, the NCG explored piloting a bespoke aHTA method(28)(29) to evaluate ten common cancer treatments that could inform the development clinical guidelines.

## Methods

We created a bespoke aHTA method by combining two existing strategies(29)—a rapid review of the literature and a ‘de facto’ HTA that included price benchmarking analysis. In addition, we estimated annualized treatment costs. The approach was based on the standard HTA process, but with necessary adaptations (30), and also drew from adaptive or rapid review processes used in other settings(29, 31–33).

We constituted a technical aHTA working group of four health economists from the International Decision Support Initiative (iDSI)(34), and seven oncologists from the NCG familiar with HTA. This group conducted the first ten oncology aHTAs, whilst making iterative adjustments throughout the process.

The aHTA process included topic selection and prioritization, scope development, evidence review, which consisted of data extraction, price benchmarking analysis, and annual drug cost calculation, then an appraisal to decide on the likely cost-effectiveness of the intervention(28). Our detailed methods are documented in the “NCG aHTA process manual”(28).

### Topic selection and prioritization

A rapid process was used to identify, select, and prioritize potential interventions for aHTA based on clinician requests for inclusion in the health benefits package. The priority was to identify the interventions for common cancers that were the least likely to be cost-effective, and which had sufficient international evidence to conduct an assessment. To inform topic selection and prioritization, we compiled a table of all interventions and a summary of background information, including treatment landscape, disease prevalence, drug prices (from the NCG’s hospital price list), and expert opinions on disease severity and equity concerns. A rapid search confirmed the availability of relevant international HTAs and cost-effectiveness analysis. The working group selected each aHTA topic through group consensus based on the evidence gathered and the NCG’s priorities.

### Development of a scope

The PICO (Population, Intervention, Comparator, Outcome) framework (35, 36) was used to develop a scope for each analysis. The scoping process fully defined the doses of the intervention and the comparators, the indication, the line of therapy, any disease-specific attributes, and outcomes of interest (Table 1, Table 3). NCG clinicians designed the final research question to reflect the local context and represent the current standard of care in India.

To ensure relevant international evidence was available, we conducted a rapid, targeted search(37–39) of established HTA agency websites, the Tufts Cost-Effectiveness Analysis registry, and the peer-reviewed literature to create a list of the available HTA reports and economic evaluations that addressed the same decision problem.

HTA agencies that were reviewed, included the National Institute for Health and Care Excellence (NICE), the Canadian Agency for Drugs and Technologies in Health (CADTH), the National Centre for Pharmacoeconomics (Ireland) (NCPE), the Pharmaceutical Benefits Scheme (Australia) (PBS) and the Pharmaceutical Management Agency (New Zealand) (PHARMAC) chosen for their high quality of analysis and freely accessible reports. HTA reports were only used if they had the same intervention, comparator(s), dose, indication and population.

To address the predominance of high-income settings in this list, economic evaluations from lower-income contexts were considered a valuable additional resource to better understand factors affecting cost-effectiveness when resources are more constrained. Where possible, evidence from India was included. The search for these studies was conducted through the Tufts CEA Registry and MEDLINE using a combination of the terms “intervention” (e.g., generic drug name) AND “indication” (e.g., type and stage of cancer), AND “economic evaluation” (to specify a cost-effectiveness analysis). Analyses were selected that aligned exactly with the PICO criteria and were from a similar economic context based on country income level and/or geographic region.

If sufficient evidence was lacking—meaning no relevant decisions from HTA bodies or cost effectiveness analysis studies were found or the evidence was considered unusable—the topic was referred for assessment via a full HTA process.

Finalizing the scope was an iterative process. Clinicians from the working group provided broad information on the intervention, which together with the details of the sourced reports informed the final decision problem.

### Data extraction

**Figure 1:**
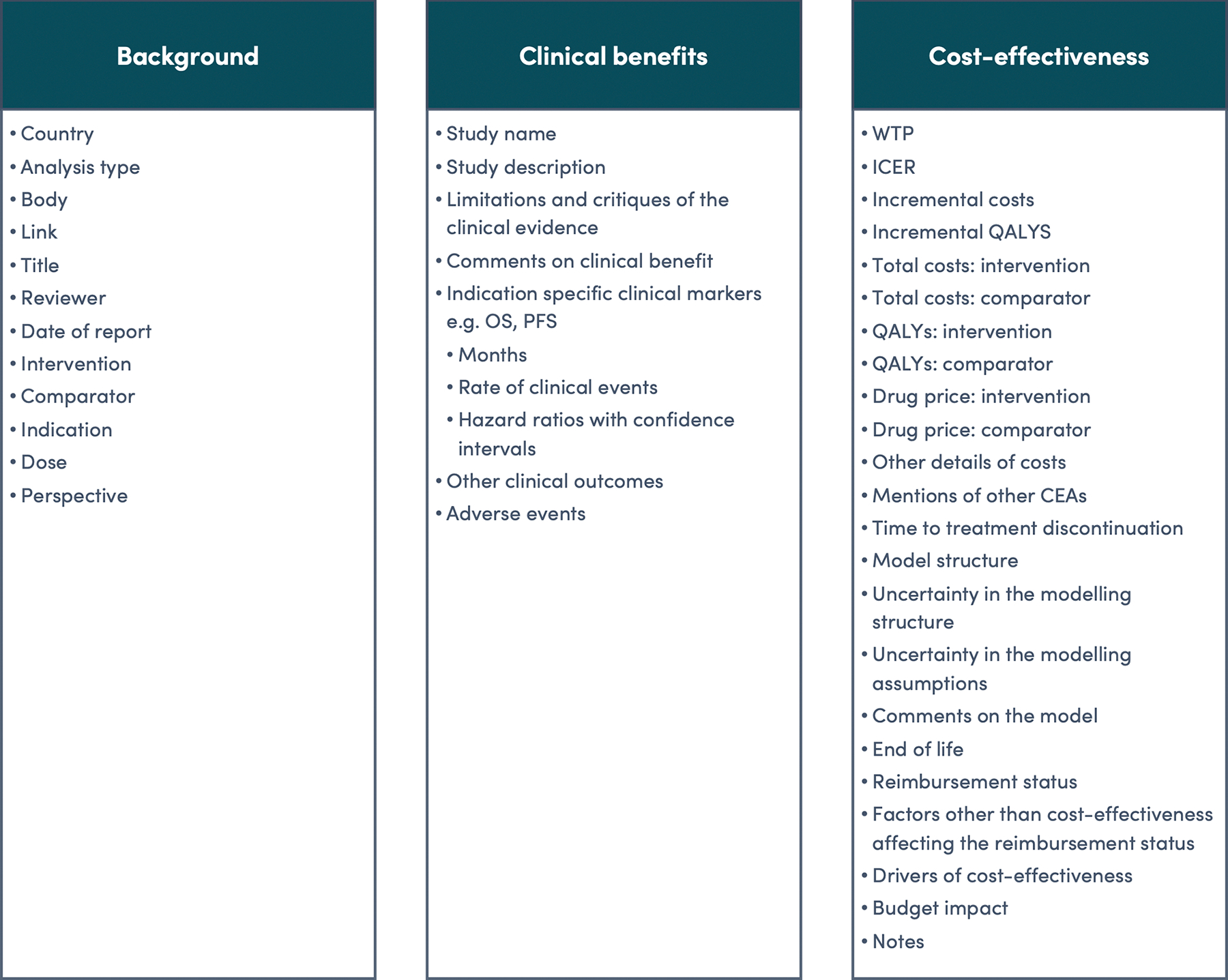
Data extraction template Abbreviations: CEA: cost-effectiveness analysis; ICER: incremental cost-effectiveness ratio; OS: overall survival; PFS: progression-free survival; QALYs: quality adjusted life years; WTP: willingness-to-pay

After developing the scope, data extraction was conducted on the available international evidence regarding the background, safety, clinical benefits, and cost-effectiveness (Figure 1). The objective was to ascertain the technology’s potential cost-effectiveness, check consistency across settings, and identify differences and uncertainties in the evidence across contexts.

The data extraction fields were drawn from the Consolidated Health Economic Evaluation Reporting Standards 2022 (CHEERS 2022)(40) checklist to evaluate reporting completeness, supplemented with additional fields for broader context. The first four aHTAs extracted top-line clinical and cost-effectiveness data, while subsequent aHTAs included additional details to broaden the evidence base for decision-making. This process identified potential sources of uncertainty, particularly those that could affect the generalisability or transferability of the evidence to India.

### Price benchmarking analysis

To supplement the evidence review, a comparative price benchmarking analysis was conducted using a published methodology(29). The objective was to compare the list price in India with that in a benchmark country, accounting for currency conversion and gross domestic product per capita adjusted for purchasing power parity (GDP PC PPP).

The GDP PC PPP adjustment was conducted because the list price for drugs in India may appear similar to the prices paid abroad once adjusted for currency, but they could still be considered less affordable if India’s purchasing power is lower than that of the benchmark country.

**Figure 2:**
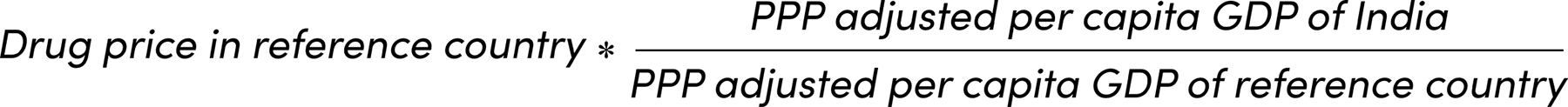
Price benchmarking formula Source: Lopert et al 2013(29)

The method converts the Indian price into the benchmark country’s currency using conversion rates, then divides this converted price by the price in Figure 2. This value represents the benchmark country’s price, adjusted by multiplying it by the adjustment factor. The adjustment factor is calculated by dividing India’s GDP PC (PPP adjusted) by that of the benchmark country. If the adjustment factor is less than 1, the price in India is lower than the benchmark country; if above 1, India is paying more.

### Annualised treatment cost

The availability of drug prices for the pharmaceutical technologies enabled the estimation of the potential annual drug costs per patient (excluding factors such as wastage, administration costs, other resources or the wider cost of illness). The objective of this calculation was to understand the potential cost impact of introducing the new technology under the AB-PMJAY scheme by determining the difference in drug costs between the intervention and comparator. Annual drug costs were determined based on the list price, pack size, dose and number of cycles. If a generic version was available, the analysis was repeated with generic prices. Drug prices were sourced from the NCG centres by co-authors, using the price paid by the hospital at the time of the analysis. A full budget impact analysis was not feasible due to data, time, and resource limitations.

Treatment costs were also quantified as the potential fraction of the family AB-PMJAY allowance, as the AB-PMJAY scheme reimbursement for secondary or tertiary healthcare is limited to ₹500,000 per family per year(13).

### Appraisal, generalisability and recommendations

The evidence from the data extraction, price benchmarking analysis and annualised drug cost calculations was appraised through group deliberation. The group assessed the evidence as a whole, considered its generalisability to the Indian context, and highlighted any transferability concerns and uncertainties, particularly variations in analyses from resource-constrained settings. The group also discussed any additional considerations outside of cost-effectiveness which would be important to highlight such as a higher disease burden or unmet need.

A recommendation was made by group consensus by placing the intervention into one of four categories: “potentially cost-effective”, “potentially not cost-effective”, “potentially cost-effective for specific subgroups” or “full HTA required”. No formal threshold was applied, however, interventions with costs far exceeding the annual family AB-PMJAY allowance with limited potential benefits were deemed “potentially not cost-effective”. Conversely, those within the allowance and offering substantial clinical benefits or were comparable to an approved treatment were deemed “potentially cost-effective”. A high level of confidence was required for either of these decisions. If the decision was uncertain, then a “full HTA was required”. If the evidence showed limited benefits for the overall population but significant benefits for a subgroup, and the costs were not excessive, then the intervention could be deemed “potentially cost-effective for specific subgroups”.

These recommendations would inform the guideline development group in deciding treatment inclusion for clinical guidelines, but would not solely determine entitlements (18).

Finally, a policy brief was produced for each case study, summarising the evidence from the rapid review process, the price benchmarking analysis results, the annual drug cost calculations, additional considerations, and the intervention’s likely cost-effectiveness designation.

### Methodological adaptations

As the aHTAs were completed, methods were iteratively refined. Additional criteria were added to the data extraction table for a more comprehensive view of the HTAs and cost effectiveness analysis reviewed. Peer-reviewed literature from similar contexts was included to strengthen the evidence base. The treatment cost calculator was adapted to compare treatment costs with the yearly family allowance under the national public health insurance scheme. Reporting in the policy briefs was expanded to improve transparency and replicability.

### Patient and Public Involvement

No patients were involved in this study.

## Results

Ten cancer interventions were deemed suitable to assess through aHTA (see Table 1), including breast cancer (n = 4), lung cancer (n = 3), head and neck cancer (n = 2) and prostate cancer (n = 1). Three aHTAs were potentially cost-effective (one after a discount and another by using the generic price); five were not, and one was only cost-effective in a subgroup. A full HTA was recommended for one intervention due to uncertainty. Eight aHTAs reviewed pharmacological technologies, while two assessed the applicability of the aHTA method to non-pharmaceutical interventions.

### Results of the data extraction

Of the ten aHTAs, sufficient international evidence was found and extracted for nine, but aHTA 8 would benefit from a full HTA due to uncertainty in the evidence. Table 1 in the Supplementary Appendix outlines the availability of crucial decision-making fields for each aHTA and the sources of evidence used; however, the content of the data was more important to decision-making than its mere availability.

For non-pharmaceutical interventions (aHTAs 5 and 6), a lack of available HTA evidence led to the introduction of peer-reviewed published literature to supplement the evidence base. However, without an economic evidence base, the method proved unsuitable for assessing non-pharmaceutical interventions. Economic evaluations continued to be included for pharmaceutical interventions as they provided valuable insight into the potential cost-effectiveness in lower income settings relevant to India.

### Results of the price benchmarking analysis

Price benchmarking analysis revealed that India commonly pays 2–4 times more than other countries for the same drug and dosage, when adjusted for currency and gross domestic product per capita adjusted for purchasing power parity (Table 2). Only one instance showed India paying less than the benchmarked country. The results suggest further discounts are required for cost-effectiveness in India.

### Results of the annualised treatment cost

Local drug costs were estimated for all pharmaceutical aHTAs (Table 3, Supplementary Appendix Table 2 and 3). Treatment costs for non-pharmaceutical interventions could not be calculated due to insufficient data available on resource use and costs. Annual drug costs for the eight pharmaceutical aHTAs ranged from 37% to 1,322% of the yearly AB-PMJAY family allowance. The treatment cost often exceeded the full annual AB-PMJAY family allowance. All interventions had higher associated treatment costs than their comparators.

### Key considerations for aHTA

Clinical benefits were the most consistently available data across all aHTAs(42) and were generally considered generalizable to the Indian population based on clinical expert feedback, with no interventions expected to have different treatment effects in India. Clinician involvement was crucial in assessing transferability, capturing standard practices in India, and identifying limitations in underlying studies.

Estimates of cost-effectiveness from international studies were less generalizable to India due to differences in prices, resource use and local considerations. The high willingness to pay threshold used by most international agencies made HTA recommendations less applicable to India, where the willingness to pay is still being defined and unlikely to match high income countries. Nevertheless, understanding the drivers of cost-effectiveness in other settings was informative to see if similar drivers applied in India. Cost-effectiveness data were crucial for highlighting uncertainties, determining if decisions were borderline cost-effective, and identifying additional considerations. Negative recommendations from high-income agencies like NICE often led to price negotiations or subsequently led to further negative recommendations by other international HTA bodies(43). Building capacity for conducting and interpreting HTA by involving experienced health economists and training clinicians to understand health economic evidence was essential for implementing aHTAs.

Results indicated that many HTA agencies had significant commercial discounts and cost offsets due to changes in resource use that were not replicable in India. Understanding clinical benefits, uncertainties, and main drivers of cost-effectiveness provided crucial insights for decision-making.

Considerations outside of cost-effectiveness, such as higher disease burden, unmet need, equity, and resource applications, were noted for policy briefs but did not impact recommendations. Following the appraisal, recommendations were made on whether the intervention was likely to be cost-effective in India, categorized as: “potentially cost-effective,” “potentially not cost-effective,” “potentially cost-effective for specific subgroups,” or “full HTA required.” Decisions never relied on a single data point but considered the entire evidence base available through the aHTA process.

## Discussion

### Overview

No single institution can feasibly undertake full HTAs for all potential interventions of interest and keep pace with the development of new pharmaceutical technologies. When traditional methods of generating economic evidence are impractical, a pragmatic approach is necessary to adapt the HTA process and strengthen the evidence base for decision-making (25).

Performing ten aHTA analyses on cancer interventions in one year saved time and resources compared to the traditional HTA process. This approach enabled more interventions to be pragmatically assessed in the time it takes to conduct one HTA, increased the economic evidence base for decision-making, and conserved resources for conducting full HTAs only for high-priority technologies with significant uncertainty or marginal cost-effectiveness.

### Suitability of interventions for aHTA

The NCG found that aHTA was most suitable for reviewing interventions where there was ample evidence, clear value for money or a need for an urgent answer. However, it was less suitable for interventions where the cost-effectiveness was ambiguous. Through this pilot, the aHTA process revealed that many requested health benefits package interventions had very high costs, often exceeding the annual family AB-PMJAY allowance and were unlikely to be considered affordable in the Indian context. An additional benefit of the aHTA process was that they rapidly generated a dossier of evidence on value for money, known as the policy briefs, which could help explain to clinicians and patients why certain interventions were considered unaffordable and could not be an entitlement under the AB-PMJAY.

While the aHTA methods proved to be faster and more efficient, further research is necessary to formalize aHTA guidelines to improve its uptake and effectiveness(26).

### Data availability and generalisability

Our methods intentionally did not include data generation or use local data, as this novel approach was designed to aggregate rapid and reliable evidence that did not require additional validation given the time and resource constraints. Drug costs were the only local data used, as they could be integrated and generalised nationally with a high level of confidence and indicated the cost impact of introducing the technology. In the future this could be adapted as cost and other data sources in India expand(44,45) to address the issue that current research is currently heavily skewed towards high-income countries (46).

### Pragmatic adaptations to the HTA process

The pragmatic adaptations to develop the aHTA approach still had the goal of assessing evidence in a robust, systematic and transparent manner. The adaptations made were assessed to see if they could be fit for purpose without introducing excessive uncertainty. Although conducted rapidly, the review’s specific scope ensured that only relevant reports were included. The price benchmarking analysis provided global context to the prices India was paying. The cost impact of an intervention was especially insightful for determining affordability. Confidence in the data was strengthened by using reports from reputable agencies like NICE. A resource-constrained decision-maker such as the NCG cannot generate and interrogate economic evidence with the same rigor as NICE, and any such analysis would be a duplication of efforts and publicly available information. By leveraging this data instead of developing new systematic reviews or economic models, the NCG could conserve time and resources and direct efforts toward focused assessment and appraisal.

### Limitations

Conducting the ten aHTAs posed several inherent challenges. First, the aHTA method described here cannot be implemented for treatments with no evidence available or where there are any deviations to the research question such as a different dose, population or line of therapy. It has limited value for non-pharmaceutical interventions or drugs that have not been assessed through HTA. However, for most pharmaceutical interventions particularly in oncology, substantial evidence was available and, aHTA was well suited to provide timely insight into the value for money of some very expensive drugs. Potentially aHTA can aid in ‘topic prioritisation’ within the HTA process, filtering out highly cost-ineffective interventions (47). This approach saves time and resources for conducting full HTA on interventions that are not well studied or have inconclusive evidence.

Secondly, while rapid data generation methods like aHTA provide pragmatic solutions, they raise concerns about regarding transparency, credibility and reliability (48). Public health finances are limited so spending decisions should be informed by robust evidence given the associated opportunity costs. Although aHTA can facilitate quicker decision-making, its findings must be interpreted cautiously, with efforts to validate results when possible. The aHTA method necessitates weighing the trade-off between the speed of decision-making against the need for certainty and confidence in the result. For example, aHTA is suitable to exclude an expensive treatment with minimal benefit from a benefits package, but requires more caution when these aspects are less clear.

Thirdly, price benchmarking analysis is a relatively crude method that benchmarks against list prices as opposed to contractual prices. List prices are often not the price included in the contract given confidential discounts, external reference pricing, parallel trade, patient access agreement and innovative contracting methods. Nevertheless, this method offers an initial insight into the affordability of the intervention in India which is useful to consider (with caution) alongside other analyses.

Finally, there are specific limitations of conducting a pilot study. This study was experimental in nature and the methods are not established and still in development. The initial aHTAs were not suitable for decision-making, however, the refinement of methods with each iterative aHTA analysis allowed them to develop in response to real world implementation challenges which rendered the methods more workable in practice. The methodology was developed in an absence of internationally recognised best practices for conducting aHTA but future developments should aim to align with the global standards as they become available.

## Conclusion

HTA evidence remains the most rigorous tool for priority-setting and countries are urged to develop their HTA systems further. However, when generating HTA evidence within limited time and resources is not feasible, aHTA serves as a reasonable alternative to using no economic evidence at all. We found that aHTA is a viable alternative to inform decision-making in resource-constrained settings when ample international data on cost-effectiveness is available. The pilot conducted on ten oncology aHTAs established a sufficient economic evidence base for evaluation, and the insights gained may contribute to the global discourse on best practices in aHTA.

## Supplementary Material

Supp1

## Figures and Tables

**Table 1:** List of assessments

AHTA	Intervention	Comparator	Disease area	Indication	Crude rate in India per 100,000(41)	Prices used by the: NCG at time of aHTA	Likely to be cost-effective in India
1	Pembrolizumab	Platinum doublet chemotherapy	Lung cancer (NSCLC)	First line, previously untreated PD-L1-positive (PD-L1 >=50%) metastatic non-small-cell lung cancer (NSCLC)	7	100 mg List price: ₹190,000	No
2	Palbociclib in combination with letrozole	Chemotherapy	Breast cancer	First line, previously untreated, hormone receptor positive, HER-2 negative, locally advanced or metastatic breast cancer	15 (In women 29.9)	25 mg List price: ₹73,920 Discounted price: ₹41,500	Not at the recommen ded price, but yes with a sufficient discount
3	Palbociclib in combination with fulvestrant	Chemotherapy	Breast cancer	Metastatic hormone receptor positive HER-2 negative breast cancer who have progressed on hormone therapy	15 (In women 29.9)		No
4	Trastuzumab in combination with paclitaxel or docetaxel	Chemotherapy	Breast cancer	First line previously untreated, hormone receptor positive or negative, HER-2 positive metastatic breast	15 (In women 29.9)	440 mg - Generic price: ₹14,000 List price: 54,000	Not at the full price but the generic price appears to be cost-effective
5	Moderate or ultra hypofractionated radiation	Adjuvant radiotherapy (normo-fractionated)	Breast cancer	For adults with non-metastatic breast cancer who have undergone mastectomy or breast conservation and optimal systemic therapy	15 (In women 29.9)	-	Yes
6	Robotic surgery (Da Vinci System)	•Open surgery •Laparoscopic surgery	Prostate cancer	Robotic surgery for prostatectomy	5.7	-	No
7	Osimertinib	• Gefitinib • Erlotinib	Lung cancer (NSCLC)	First-line, previously untreated EGFR mutated metastatic NSCLC	7	80 mg 10 tabs per pack 3 packs in a box. Cost of the box ₹439,478	No
8	Ceritinib	• Crizotinib	Lung cancer (NSCLC)	First-line, previously untreated ALK positive, metastatic, NSCLC	7	10 tablets of 150 mg ₹6,736	Full HTA needed
9	Nimotuzumab with radiotherapy	Radiotherapy with cisplatin	Head and neck cancer	Newly diagnosed, treatment-naive adult patients with stage iii or iv locally advanced head and neck squamous cell carcinomas (LAHNSCC) who were fit for radical chemoradiation	13.4	200 mg ₹44,352.00	No
10	Cetuximab with radiation	Radiation alone	Head and neck cancer	Newly diagnosed, treatment naive, non-metastatic patients with stage iii or iv locally advanced head and neck squamous cell carcinomas (LAHNSCC)	13.4	1000 mg ₹15,979	Not for all patients but potentially cost-effective for a subgroup based on efficacy data

**Table 2:** Price benchmarking results

aHTA	NCG price at time of aHTA (INR)	Benchmark country, currency and GDP PC PPP	Benchmark country price from the data extraction	Adjustment Factor (India GDP PC PPP of $7,034/ benchmark country GDP PC PPP)	Currency converter rate	Indian price converted to the benchmark country price	Price paid by the benchmark country when adjusted for GDP PC PPP	Price Ratio
1	190,000	UK (GBP) $48,710	5,260	0.1444	0.0102	1944	760	2.56
		New Zealand (NZD) $43,953	8,000	0.1600	0.0203	3859	1280	3.01
		USA (USD) $65,281	9,724	0.1078	0.0134	2545	1048	2.43
2 & 3	41,500	UK (GBP) $48,710	2,905	0.1444	0.0102	423	419	1.01
		New Zealand (NZD) $43,953	4,000	0.1600	0.0203	842	640	1.32
		Australia (AUD) $53,320	4,265	0.1319	0.0187	776	563	1.38
		USA (USD) $65,281	13,007	0.1078	0.0134	556	1401	0.40
7	439,478	Ireland (EUR) $93,612	6,200	0.0751	0.0120	5274	466	11
		UK (GBP) $44,916	5,770	0.1566	0.0098	4307	904	5
8	101,040	UK (GBP) $44,916	4,923	0.1566	0.0098	990	771	1.28
9	439,478	Ireland (EUR) $93,612	6,200	0.0751	0.0120	5274	466	11
10	159,79	UK (GBP) $44,916	178	0.1566	0.0097	155	27.89	6

N.B. Insufficient evidence was available to conduct price benchmarking on aHTAs 4–6. The currency exchange rates were sourced through Google Finance for the date of the aHTA using the average 1 year rate.

Abbreviations: GDP PC PPP: Gross domestic product per capita purchasing power parity; INR: Indian rupees

**Table 3:** Calculated treatment costs

AHTA	Intervention		Cost/pack	Cost /dose	Cost/cycle	Annual cost	% of family AB-PMJAY allowance ₹500,000
1	Pembrolizumab		₹ 190,000	₹ 380,000	₹ 380,000	₹ 6,609,286	1,322%
2	Palbociclib		₹ 41,500	₹ 1,976	₹ 41,500	₹ 541,353	108%
	Letrozole		₹ 350	₹ 7,000	₹ 7,000	₹ 105,000	21%
3	Palbociclib		₹ 41,500	₹ 1,976	₹ 41,500	₹ 541,353	108%
	Fulvestrant		₹ 30,800	₹ 61,600	₹ 61,600	₹ 924,000	185%
4	Trastuzumab		₹ 14,000	₹ 8,909	₹ 8,909	₹ 115,818	23%
7	Osimertinib		₹ 439,478	₹ 14,649	₹ 439,478	₹ 5,350,645	1,070%
	Gefitinib		₹ 14,000	₹ 467	₹ 14,000	₹ 170,450	34%
	Erlotinib		₹ 56,000	₹ 1,867	₹ 56,000	₹ 681,800	136%
	Difference				Gefitinib Erlotinib	+₹ 5,180,195 +₹ 4,668,845	
8	Ceritinib		₹ 6,736	₹ 2,020	₹ 61,508	₹ 738,097	148%
	Crizotinib		₹ 87,000	₹ 1,450	₹ 44,134	₹ 529,613	106%
	Difference					₹ 208,485	
9	Nimotuzumab		₹ 44,352	₹ 44,352.00	₹ 44,352	₹ 310,464	62%
10	Cetuximab	₹ 15,979	₹ 73,902.88	₹ 73,903	₹ 591,223	118%	

## Data Availability

All data used within this analysis were from publicly available sources and can be provided upon suitable request.

## Key messages

### What is already known on this topic:

There is a pressing need for more evidence-informed priority-setting. However, implementation often faces substantial challenges, including limited data, capacity, time, and resources. To address these issues, some working groups employ rapid or adaptive health technology assessment (HTA) methods.

### What this study adds:

One of the first examples of adaptive HTA being trialled in a real world setting and the insights and lessons learned from such a pilot programme.

### How this study might affect research, practice, or policy:

The aHTA method could be formally implemented into the process of reviewing the entitlements under the Indian national health insurance scheme. This would increase the level of economic evidence used in decision-making which may lead to a more efficient and sustainable health benefits package. Insights from these ten aHTA evaluations may also contribute to the global dialogue on the development and use of aHTA methods.
