# Supplementary material for: Exploring Adaptive Health Technology Assessment for Evaluating Ten Cancer Interventions: Insights and Lessons from a Pilot Study in India
Source: BMJ Evid Based Med. Author manuscript; Available in PMC 2025 Nov 27. (PMC12213987; doi:10.1136/bmjebm-2023-112490)
Supplement: Supp1 [file NIHMS2045019-supplement-Supp1.pdf]

Supplementary Appendix

This Supplementary Appendix has been provided to supply readers with additional information about the following work:

Adaptive health technology assessments: results for ten cancer interventions in India

Srobana Ghosh[1], C S Pramesh[2] Manju Sengar[2], Priya Ranganathan[2], Francis Ruiz[3], Tabassum Wadasadawala[2], Prakash Nayak[2], Jayashree Thorat[2], Apurva Ashok[2], Malkeet Singh[1], Abha Mehndiratta[1], Cassandra Nemzoff[1,3] and Hiral Anil Shah[1,]

Affiliations

1. Center for Global Development, London, UK
2. Tata Memorial Centre, Homi Bhabha National Institute, Mumbai, Maharashtra, India
3. London School of Hygiene and Tropical Medicine, UK

Table 1: Information aggregated from the aHTA process by analysis

aHTA	Key data points					Source of evidence used in the data extraction		
	Clinical outcomes	ICER	Inc. £	Inc. QALYs	Decision	HTA appraisal	Systematic review	CEA study
1	●	●	-	-	●	●	-	-
2	●	●	-	-	●	●	-	-
3	●	●	-	-	●	●	-	-
4	-	-	-	-	●	●	-	-
5	●	-	-	-	-	-	●	-
6	-	●	●	●	●	●	●	●
7	●	●	●	●	●	●	-	●
8	●	●	●	●	●	●	-	●
9	●	●	●	●	●	-	-	●
10	●	●	●	●	●	●	-	●

Abbreviations: aHTA: adaptive health technology assessment; CEA: cost-effectiveness analysis; HTA: health technology assessment; ICER: incremental cost-effectiveness ratio; Inc: incremental; QALY: quality adjusted life years

Legend: – information not available; ● information available

Table 2 *Intervention doses and treatment cost calculation inputs*

AHTA	Intervention	(A) Days in cycle	(B) Cycles	(C) Units/pack	(D) Mg/unit	(E) Cost/pack	(F) Mg/dose	(G) Doses / cycle
1	Pembrolizumab	21	17	1	100	₹ 190,000	200	1
2	Palbociclib	28	13	21	125	₹ 41,500	125	1
	Letrozole	30	15	10	2.5	₹ 350	500	1
3	Palbociclib	28	13	21	125	₹ 41,500	125	1
	Fulvestrant	30	15	1	250	₹ 30,800	500	1
4	Trastuzumab	7	13	1	440	₹ 14,000	4 mg/kg	1
7	Osimertinib	30	12	30	80	₹ 439,478	80	30
	Gefitinib	30	12	30	250	₹ 14,000	250	30
	Erlotinib	30	12	30	150	₹ 56,000	150	30
8	Ceritinib	30	12	10	150	₹ 6,736	450	30
	Crizotinib	30	12	60	250	₹ 87,000	250	30
9	Nimotuzumab	7	7	1	200	₹ 44,352	200	1
10	Cetuximab	7	8	1	100	₹ 15,979	462.5	1

Table 3: *Treatment cost calculation results*

AHTA	Intervention	(H) Cost/mg (E÷(C*D)	(I) Cost /dose H*F	(J) Cost/cycle G*I	(K) Annual cost J*B	(L) % of family AB-PMJAY allowance K÷₹500,000
1	Pembrolizumab	₹ 1,900.00	₹ 380,000	₹ 380,000	₹ 6,609,286	1,322%
2	Palbociclib	₹ 15.81	₹ 1,976	₹ 41,500	₹ 541,353	108%
	Letrozole	₹ 14.00	₹ 7,000	₹ 7,000	₹ 105,000	21%
3	Palbociclib	₹ 15.81	₹ 1,976	₹ 41,500	₹ 541,353	108%
	Fulvestrant	₹ 123.20	₹ 61,600	₹ 61,600	₹ 924,000	185%
4	Trastuzumab	₹ 31.82	₹ 8,909	₹ 8,909	₹ 115,818	23%
7	Osimertinib	₹ 183.12	₹ 14,649	₹ 439,478	₹ 5,350,645	1,070%

	Gefitinib	₹ 1.87	₹ 467	₹ 14,000	₹ 170,450	34%
	Erlotinib	₹ 12.44	₹ 1,867	₹ 56,000	₹ 681,800	136%
	Difference			Gefitinib<br>Erlotinib	+₹ 5,180,195<br>+₹ 4,668,845	
8	Ceritinib	₹ 4.49	₹ 2,020	₹ 61,508	₹ 738,097	148%
	Crizotinib	₹ 5.80	₹ 1,450	₹ 44,134	₹ 529,613	106%
	Difference				₹ 208,485	
9	Nimotuzumab	₹ 221.76	₹ 44,352.00	₹ 44,352	₹ 310,464	62%
10	Cetuximab	₹ 15,979	₹ 73,902.88	₹ 73,903	₹ 591,223	118%

Table 4 *Drivers of cost-effectiveness*

AHTA	Likely to be cost-effective in India	Recommended in other jurisdictions			Drivers of cost-effectiveness			
		**Yes**	**No**	**Benchmark country has a high willingness to pay threshold and/or commercial discount**	**Intervention associated with a significant clinical benefit with reasonable confidence**	**Intervention associated with an uncertain or small clinical benefit**	**Intervention saves costs or resources**	**Intervention associated with very high costs**
1	No	• England<br>• USA	• New Zealand	✓	✓			
2	Not at the recommended price, but yes with a sufficient discount	• Australia<br>• England<br>• New Zealand<br>• Thailand<br>• USA	-	✓	✓			
3	No	• England (CDF)<br>• New Zealand<br>• USA	• Australia	✓		✓		
4	Not at the full price but the generic price appears to be cost-effective	• Australia<br>• England<br>• USA<br>• New Zealand	-	✓	✓			
5	Yes	• No HTA Agency has appraised the CEA but multiple studies found that it could be	• -				✓	

		cost-effective						
6	No	• USA	• Australia- Potentially<br>• Canada – Potentially<br>• Canada			✓		✓
7	No	• England<br>• Ireland<br>• WHO – Yes when no feasibility, cost, or affordability constraints limit the access	• Canada<br>• Singapore	✓		✓		✓
8	Full HTA needed	• Canada<br>• China<br>• England	•	✓		✓		
9	No	• China – Yes (but not cost-effective)	•			✓		✓
10	Not for all patients but potentially cost-effective for a subgroup based on efficacy data	• England – Yes for a subgroup of patients	•	✓		✓		
